# CX3CR1 Receptor Polymorphisms, Th1 Cell Recruitment, and Acute Myocardial Infarction Outcome: Looking for a Link

**DOI:** 10.1155/2013/451349

**Published:** 2013-11-07

**Authors:** S. Pucci, P. Mazzarelli, M. J. Zonetti, T. Fisco, E. Bonanno, L. G. Spagnoli, A. Mauriello

**Affiliations:** Department of Biomedicine and Prevention, University of Rome “Tor Vergata”, Via Montpellier 1, 00133 Rome, Italy

## Abstract

Fractalkine is a proinflammatory chemokine that participates in atherosclerotic process mediating the interactions of vascular cells and leukocytes and selective recruitment of Th1 lymphocytes, through interaction with CX3CR1 receptor. The polymorphism of the fractalkine receptor 280M-containing haplotype, which codifies for a receptor with minor expression and with a reduced binding capability, represents a novel protective factor of atherosclerotic disease. We investigated the association among CX3CR1 genotype, the inflammatory infiltrate subpopulations recruited in the plaque, and the in situ expression of fractalkine and its receptor, in patients who died of myocardial infarction (AMI) compared with subjects who died of noncardiac causes. Patients with nonlethal AMI (AMI survivors) were also investigated to correlate the CX3CR1 polymorphisms and the incidence of lethal AMI. A strong T cells infiltrate was found in infarct related artery (IRA) plaques of AMI patients presenting the V_249_ T_280_ haplotype (84%). Conversely, a decreased T cell recruitment was associated with I_249_T_280_ haplotype in the controls (64%). The significant higher presence of the variant allele I249 in homo- and heterozygosis, found in controls (91%) and in AMI survivors (94%), with respect to the patients who died of AMI (48%), showed the relevance of this polymorphism both in the onset and outcome of acute myocardial infarction. The presence of CX3CR1 polymorphisms could influence the incidence and the outcome of acute myocardial infarction, altering the inflammation of the whole coronary tree by the impaired recruitment of Th1 polarized subpopulation in the coronary plaque.

## 1. Introduction

Genetic factor appears to be important determinant of cardiovascular disease. In particular, specific genetic polymorphisms that modulate blood pressure, coagulation, and lipid metabolism have been identified to be associated with risk of coronary artery disease. However, none of these associations has identified the precise molecular basis of pathological accumulation of leukocytes in vessel wall that characterized the onset of acute myocardial infarction.

Acute myocardial infarction (AMI) in fact is related to a diffuse active inflammation of the coronary tree associated with rupture of one of the multiple vulnerable plaques.

Unlike stable plaques characterized by a chronic infiltrate of macrophages, vulnerable plaques display an inflammatory infiltrate composed by T cells activated towards a Th1 pathway, and whose recruitment is regulated by chemokines, cytokines, and their receptors [1, 2].

In particular, chemokines are part of effector and amplification mechanisms of polarized Th1 or Th2 mediated immune responses; therefore, their receptors may serve as Th1 versus Th2 marker and lead to the selective modulation of T cell dependent immunity. Hence, the receptor expression and the chemokines release are closely linked to T cell polarization and activation.

Fractalkine (CX3CL1) is a proinflammatory chemokine that induces chemotaxis of circulating monocytes and selective recruitment of Th1 lymphocytes through interaction with its ligand, the CX3CR1 receptor, that is highly expressed on Th1 activated T cells [3–11]. It has been observed that the expressions of fractalkine and its receptor CX3CR1 are upregulated in atherosclerosis lesions, and the severity of atherosclerosis is greatly improved by inhibiting their expressions, indicating that the fractalkine/CX3CR1 pair is closely related to atherosclerosis.

Studies on CX3CR1 receptor knock out animal models and on human coronaries with native atherosclerosis point out the important role of fractalkine in atherogenesis and plaque progression [12, 13].

Recent data demonstrated the enhanced expression of CXCL1 and its receptor (CX3CR1) in coronary artery disease that could be downmodulated by statin therapy [14]. These data suggest a role of CXCL1 and CXCR1 as therapeutic targets in the care of CAD patients including those with unstable disease.

Epidemiological studies have proved the existence of two common nonsynonymous single-nucleotide polymorphisms of CX3CR1 receptor gene (V249I and T280M). The polymorphisms of the fractalkine receptor, that codify for a molecule with minor expression and with a reduced binding capability, have been proposed as a novel protective marker for coronary disease. In particular, the CX3CR1 I249 allele in homo- and heterozygote conditions has been associated with a marked reduction in risk of acute coronary events [15–17]. In addition, population studies on CX3CR1 polymorphism have firmly confirmed that 280 M-containing haplotype is associated with reduced risk of atherosclerotic disease. As shown by experimental studies, the presence of the I_249_M_280_ haplotype codifies for a chemokine receptor variant that has impaired adhesive function, low binding activity, and low recruitment efficiency of T cells within the plaque. On the contrary, the CX3CR1 V_249_T_280_ haplotype, characterized by high expression and binding efficiency, is associated with high risk of CAD and could be related to plaque vulnerability and rupture.

All these data suggest that the CX3CR1 V249 and T280 polymorphisms could amplify the Th1 response triggering plaque vulnerability and rupture. Moreover, the higher expression and binding activity of this receptor could influence the atherogenesis in patients with artery disease and the incidence and the outcome of the myocardial infarction. 

Little information is available regarding the risk factors predicting the subsequent lethal cardiac events after acute myocardial infarction (AMI). In fact, despite the use of advanced medical and invasive procedures, the risk adjusted 90-day mortality rates in patients with myocardial infarction are up to 20–24%. Recent data pointed out the relevant role of diffuse inflammatory process. We have recently demonstrated, in patients who died of a first AMI, the presence of a strongly activated T cell infiltrate in the entire coronary tree and in the peri-infarctual and in the remote unaffected myocardial regions underlying both coronary and myocardial vulnerability [18, 19]. In view of that, the object of this investigation was to determine if the CX3CR1 polymorphisms, genetic factors previously described to be involved in atherogenesis, play a role in the pathobiology of acute myocardial infarction.

Therefore, we evaluated the inflammatory burden and the in situ expression of fractalkine and its receptor in coronary plaques of patients died of myocardial infarction (AMI) as compared to subjects died of noncardiac causes in order to focus if the presence of I249/M280 haplotype correlates with an impaired Th1 cell recruitment in the plaque.

We further evaluate if the CX3CR1 polymorphisms could influence the outcome of acute myocardial infarction. At this purpose, we examined the of CX3CR1 polymorphisms presence in individuals who died of noncardiac causes, in a cohort of patients that survived at acute myocardial infarction (AMI survivors) compared with patients died of AMI.

## 2. Methods

### 2.1. Case Selection

We observed 25 patients (17 males/8 females, mean age 70.5 ± 1.8 years) died of acute myocardial infarction (AMI group) and 22 age-matched patients (15 males/7 females, mean age 73.7 ± 2.8) died of noncardiac causes and without clinical cardiac history (NCC group). Further, 16 patients (mean age 65 ± 9.9 yr, 13 males) who survived at a previous myocardial infarction (MI) suitable for surgical ventricular restoration have been analyzed (SVR group). Clinical, angiographic and surgical characteristics are listed in Table 1. In the AMI group, the time from symptom onset to death was within 72 hours for all cases. Clinical history and standard electrocardiographic findings defined acute myocardial infarction. Histopathological examination of the myocardium confirmed AMI as cause of death. The infarct was transmural and from a single coronary artery distribution in all cases. Myocardium from NCC patients, died of bronchopneumonia, pulmonary embolism, and intestinal hemorrhage, did not show either infarct or necrosis in all cases.

The main indications for surgery of patients of SVR group were symptoms of heart failure, angina, and/or a combination of the two. Four patients of this group (25%) had associated mitral valve repair for a grade 3+/4+ of mitral regurgitation. The surgical technique has been previously described [1]. Mitral valve (MV) is repaired, when needed, through the ventricle.

Patients with chronic inflammatory diseases or tumors were excluded from the study to avoid bias due to immunological changes. All autopsies were performed within 12–24 hours of death. 

### 2.2. Tissue Handling and Processing

At the autopsy, in AMI and NCC, the 4 major epicardial coronary arteries (left main, left anterior descending, left circumflex, and right) were dissected from each heart, 10% buffered formalin fixed, cut transversely at 5 mm intervals, decalcified if necessary, paraffin embedded, and serially sectioned for morphological and immunohistochemical studies. The hearts were transversally cut at 1 cm intervals from the apex to the base. The myocardium was examined for the presence and extent of infarction. The location of myocardial infarction was detected by immersion of a cross-section of the heart in a solution of triphenyltetrazolium chloride (for the autoptic demonstration of myocardial infarction). In addition, in all cases, another cross-section of the heart was processed for histological examination, and the infarction was confirmed by light microscopy.

In SVR patients, LV myocardial samples were obtained at the time of surgical operation. The tissue was taken from the transition zone between MI and the remote, normal region. All tissues were fixed in 10% formalin and embedded in paraffin.

### 2.3. Morphological Studies

Coronary sections from the two groups, H&E and pentachrome stained, were examined by light microscopy. Culprit plaques were defined as lesions showing either plaque rupture or erosion with superimposed thrombus. The coronary artery showing the culprit lesion was defined as infarct-related artery (IRA). In AMI were examined: the culprit segment in the IRA, including the thrombotic lesion, 1.5 cm long, a segment 1.5 cm long in which maximum of stenosis was present in the non-IRA coronary. In NCC, a segment of 1.5 cm long in which maximum of stenosis was present was selected. All coronary segments were cut transversely at 5 mm intervals. A total of 216 arterial segments were paraffin embedded and serially sectioned: 150 from the AMI group (75 from IRA and 75 from non-IRA coronaries) and 66 from NCC group, respectively.

### 2.4. Immunohistochemical Studies (IHC)

Serial 3 *μ*m thick sections were cut from paraffin blocks and mounted on SuperFrost Plus slides (Menzel-Glaser, Germany). *α*-smooth actin, CD68, CD3 (Dakopatts, Denmark), fractalkine (R&D System, Minneapolis, MN), and CX3CR1 (GmbH Gottingen, D) primary monoclonal antibodies were used. CD68 (alkaline phosphatase/anti-alkaline phosphatase) and CD3 (DAB detected) IHC was used to quantify the inflammatory infiltrate. For each antibody, positive cells, as a mean of 10 randomly selected fields per section, were counted at a magnification of 40X in the plaque shoulder region and in the cap itself.

### 2.5. Confocal Microscopy Immunofluorescence

In order to characterize the cell subpopulations that express CX3CR1, we performed a triple immunostaining for confocal microscopy. Paraffin sections of coronary plaques were first incubated with anti-CD3 MoAb (1 : 20, 1 h at RT), rinsed, and then incubated with biotinylated mouse IgG; Fluorescence was obtained by incubating the sections with a streptavidin-coumarin fluorochrome. A second reaction with primary antibodies to CX3CR1 (dilution 1 : 50, 1 h at RT in the dark) was performed. Fluorescence was obtained with a conjugate streptavidin-fluorescein conjugate (FITC). A third immunostaining was performed by incubating the sections with anti-IFN gamma monoclonal antibody (dilution 1 : 25, 1 h at RT in the dark). Sections were incubated with tyramide signal amplification biotin system (NEN Life Science Products, Boston, MA, USA). Fluorescence was obtained with a streptavidin-Texas Red fluorescent conjugate. Control sections were incubated with a mixture of irrelevant monoclonal reagents having a similar isotype. Images were acquired by means of Noran confocal microscope at 60X/1.4 NA immersion oil lens. Three D stacks were acquired at a resolution of 0.1 micron in *X*-, *Y*-, and *Z*-axis.

### 2.6. DNA Extraction

DNA was extracted from formalin fixed and paraffin embedded heart of the subjects described above. Paraffin was removed with xylene for 5 min at room temperature. Samples were centrifuged at full speed for 5 min at RT. Supernatant was removed, and 1.2 mL of 100% ethanol was added to remove residual xylene. The DNA was extracted with the QIAamp DNA kit (Qiagen GmbH, Germany) and resuspended in 50 *μ*L of Buffer AE.

### 2.7. Screening for Polymorphisms

The DNA quality was controlled by a genomic polymerase reaction assay (PCR) using the primer set for BAK gene. The following two polymorphisms have been analyzed: I249, a guanidine to adenine substitution at nucleotide 745 of the open reading frame changing valine at position 249 to isoleucine, and M280, a cytosine to thymidine substitution at nucleotide 839 changing a threonine at position 280. The PCR product was analyzed in 2% agarose gel stained with ethidium bromide. CX3CR1 gene T280M and V249I mutations were identified after amplification of 311 base pairs (bp) (primers: forward: 5′ AGA ATC ATC CAG ACG CTG TTT TCC 3′; reverse: 5′ CAG AGG ACA GCC AGG CAT TTC C 3′). The T280M and V429I were detected on the same amplified fragment. Amplification reactions were performed using 35 cycles of 95°C, 69°C, and 72°C for 30 seconds each, preceded by a single cycle of 95°C for 3 minutes and followed by a single cycle of 72°C for 10 minutes. In order to type the alleles at codons 249 and 280, 5 *μ*L of PCR reaction was digested, respectively, with Acl I (New England Biolabs, Beverly, MA) or BsbmB1 (New England Biolabs, Beverly, MA) in 20 *μ*L of reaction as previously described [11]. In order to evaluate the efficiency of endonucleases enzymes, control DNA with Acl I and Bst 4CI restriction sites was used, and DNA was loaded at different times on agarose gel. The genotypes were analysed and determined by analytical electrophoresis of each digestion on a 2% agarose gel.

### 2.8. Statistical Analysis

Statistical analysis of the data was performed with SPSS for Windows (version 11.0, SPSS, Chicago, IL). Unpaired *t*-test was utilized to assess intergroup differences for continuous variables. The *X* ^2^ test was used to establish differences in the distribution between categorical variables. The statistical significance of different polymorphisms present in the single groups was identified by Fisher's exact test. A *P* value of <0.05 was considered statistically significant.

Using logistic regression analysis (SPSS 13.0), the association of each polymorphism with the cardiovascular events (fatal AMI, survivors, and control group) was adjusted for age, gender, smoking cigarette, hypertension, diabetes mellitus, and hyperlipidemia.

## 3. Results

### 3.1. Coronary Plaques Inflammation

The examination of the whole coronary tree of the AMI group demonstrated the presence of multiple complicated atherosclerotic plaques with a thin fibrous cap and a high inflammatory infiltrate; moreover, in the coronary branch supplying the infarct area (IRA), we found a disrupted/thrombotic plaque (culprit plaque). Conversely, the coronary trees of the control group (NCC) showed atherosclerotic lesions with a thick fibrous cap and a scanty inflammatory infiltrate. No plaque disruption or erosion or thrombosis has been observed. Clinical, autoptic, and surgical patient characteristics are showed in Table 1.

**Figure 1:**
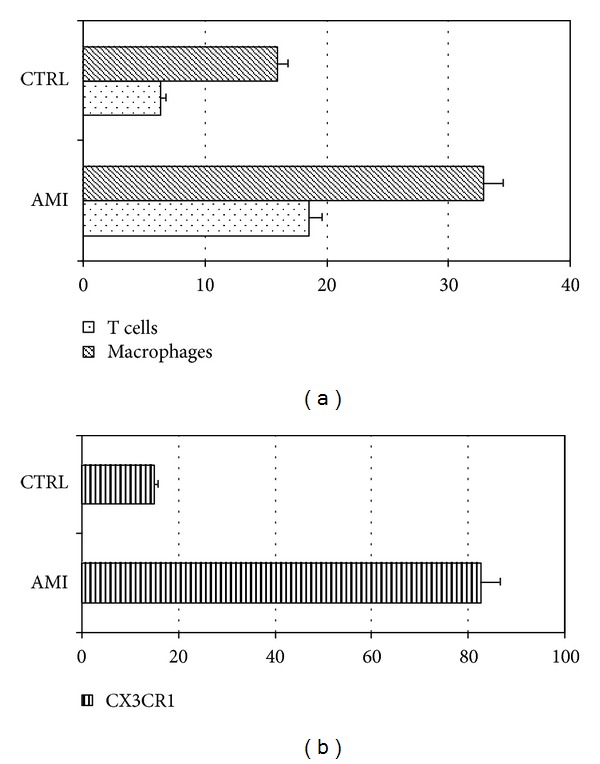
Inflammatory cells characterization. (a) shows the percentages of inflammatory cells, T lymphocytes (dotted bar) and macrophages (dashed bar) in AMI and control group. (b) Shows the percentages of T cells CX3CR1 positive in AMI and control group.

Cell subpopulations were characterized by immunohistochemical studies using specific antibodies for lymphocytes (CD3, T cells), macrophages (CD68), and smooth muscle cells (*α*-actin). A quantitative analysis demonstrated a significant (*P* < 0.01) increase in the inflammatory cells per area (CD3 and CD68 positive) in AMI plaques (51.4 ± 2.5%) as compared with the plaques of the NCC group (22.4 ± 1.9%). T lymphocytes represented the 18.5 ± 2.3% of the plaque cells in AMI and the 6.4 ± 1.8% of cells in the NCC, whereas macrophages were 32.9 ± 3.2% in the AMI and 16.0 ± 1.8% in the NCC (Figure 1(a)). Moreover, the expression of IFN-*γ* was assessed in culprit lesions by immunofluorescence staining (coronary artery from AMI group) with confocal microscopy (Figure 3).

### 3.2. Fractalkine and CX3CR1 Expression

The Fractalkine expression was evaluated by immunohistochemistry in order to compare T cell population recruitment and chemokine release.

**Figure 2:**
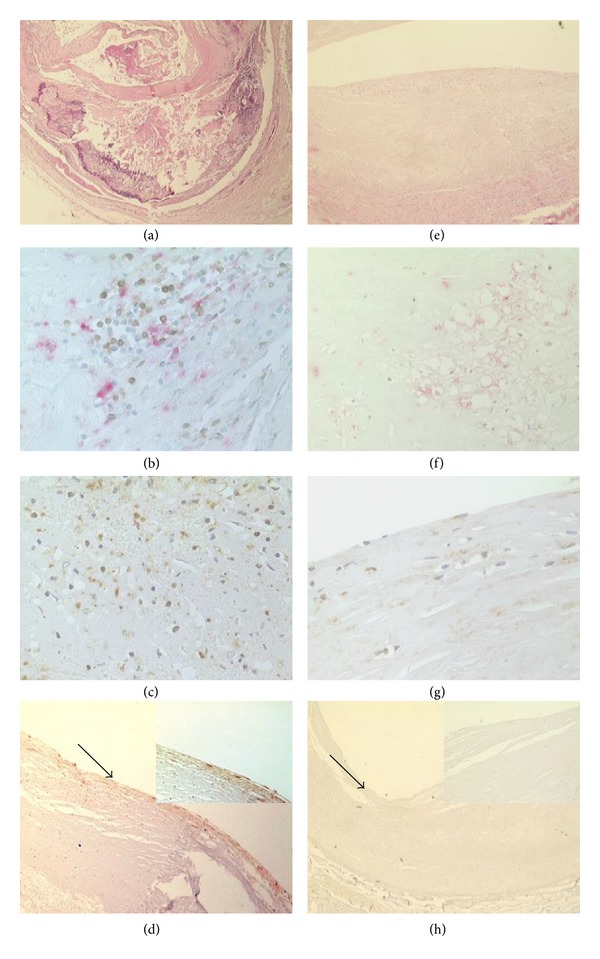
Coronary plaques morphology and inflammatory infiltrate characterization in AMI group (a)–(d) and control group (e)–(h). (a) shows an haematoxylin eosin stain of a plaque from AMI group showing a complicated plaque with a large necrotic core, a thin cap, and a high inflammatory infiltrate with a high percentage of lymphocytes, ((b) macrophages CD68 positive, revealed by alkaline phosphatase/anti-alkaline phosphatase, red reaction, and T lymphocytes CD3 positive DAB detected streptavidin/biotin immunoperoxidase, brown reaction). (c) shows the immunostaining against the fractalkine receptor CX3CR1 that gave rise to a strong diffuse positive reaction in the T cells of AMI group. (d), (in the insert a particular corresponding to the area indicated by the arrow, magnification: 40x) demonstrate a positive reaction for fractalkine in the endothelial cells. (e) shows an haematoxylin eosin of a coronary from a patient died by bronchopneumonia without clinical cardiac history. The plaque shows a thick fibrotic cap, and the inflammatory infiltrate is scanty and predominantly constituted by macrophages ((f) macrophages CD68 positive, revealed by alkaline phosphatase/anti-alkaline phosphatase, red reaction, and T lymphocytes CD3 positive DAB dectected streptavidin/biotin immunoperoxidase, brown reaction). The immunostaining for the fractalkine receptor CX3CR1 gave rise to a weak positive reaction in the smooth muscle cells and macrophages ((g) 10x magnification). The reaction for fractalkine was negative in the control group (h) (in the insert a particular, corresponding to the area indicated by the arrow, magnification 40x).

**Figure 3:**
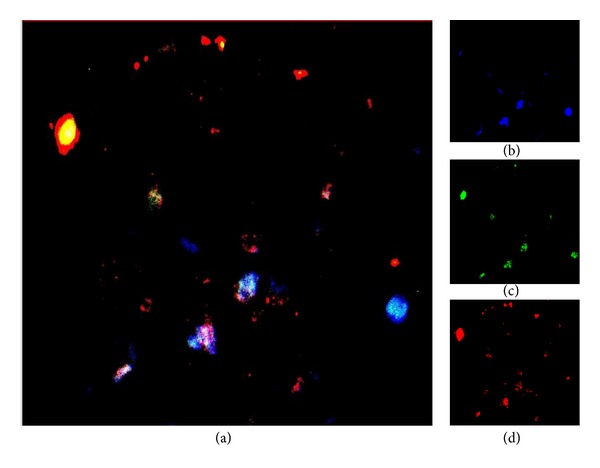
Confocal microscopy examination of triple stain in a culprit lesion (coronary artery from AMI group). (a) (magnification 2000x) shows a 2D reconstruction triple stain for T cells, CD3 (blue), fractalkine receptor CX3CR1 (green), and IFN-gamma (red); yellow, light blue, and white areas were due to multiple positivity. (b) CD3 immunostaining revealed by coumarin conjugated streptavidin; (c) CX3CR1 immunostaining revealed by FITC conjugated streptavidin; (d) IFN-gamma immunostaining revealed by Texas Red conjugated streptavidin.

Figure 2(b) shows the expression of CD3, fractalkine receptor CX3CR1 Figure 2(c) in T cells infiltrating the culprit lesion. In AMI plaques, immunostaining for fractalkine was strongly positive (Figure 2(d)). The immunofluorescence in Figure 3 demonstrated the coexpression of these three proteins in the same cells of AMI culprit plaque confirming the existence of a T cell chronic active inflammation. Moreover, these data demonstrated the Th1 mediated immune response (IFN-*γ* production) involved in plaque inflammation and destabilization correlated with the presence of Th1 polarized T cells (CXCR1 expressing cells) recruited in the plaque by the interaction of fractalkine and the CX3CR1 receptor on the T cell membrane.

The immunohistochemical studies demonstrated that endothelial cells in plaques of AMI patients gave rise to a diffuse positive reaction for fractalkine. Smooth muscle cells were focally positive, while lymphocytes and macrophages were not. Conversely, in the plaques of NCC group, all the cell types were almost negative for fractalkine (Figure 2).

The fractalkine receptor (CX3CR1) was strongly and diffusely expressed in the cell membrane in T lymphocytes of AMI plaques (Figure 2(c)) as compared to control. Macrophages and smooth muscle cells gave rise to a weak and scattered positive reaction. The reaction was almost negative in the plaques of NCC; group only a weak positive staining was present in smooth muscle cells and macrophages in the plaque (Figure 2(g)).

The quantitative study revealed that 83% of T cells in the AMI plaques were CX3CR1 positive (Figure 1(b)) indicating a Th1 subset polarized subpopulation. Furthermore, a confocal observation of IFN-*γ* expression confirmed that T lymphocytes expressed IFN-*γ* (Figure 3).

In the coronary, plaques from control group, T lymphocytes showed a lower level of CX3CR1 positivity (18% of T lymphocytes were positive) (Figure 1(b)); in this group, the reaction for IFN-*γ* was completely negative (not shown).

### 3.3. CX3CR1 Genotype Correlates with the Th1 Cells Recruitment

It has been previously demonstrated that CX3CR1 genotypes influence the fractalkine receptor binding affinity and therefore Th1 lymphocytes recruitment. In order to correlate the presence of activated Th1 lymphocytes with CX3CR1 genotype, patients were genotyped for the V249I and T280M polymorphisms of CXCR1 gene. DNA extracted from myocardial formalin fixed and paraffin embedded sections was analyzed by polymerase chain reaction. The genotype was determined on the amplicon of 311 bp after digestion with endonucleases specific for each polymorphism. The analysis of CX3CR1 V249I and T280M polymorphisms demonstrated that 13 out of 25 patients who died of infarction (52%) were homozygous for the V249 allele and 21 out of 25 (84%) were homozygous for the T280 allele (Table 2). Moreover, the 40% of AMI patients (10/25) were heterozygous for the V249 allele, but all of these were homozygous for T280 allele as shown in Table 2. On the other hand, only 8% (2/25) of AMI patients displayed I249 allele in homozygosis and T280 in homo- (84%) or heterozygosis (16%). Notably, the two AMI patients who presented the variant haplotypes 249I/I and 280T/M had reduced T infiltrate and low IFN-*γ* production in the plaque but a large number of B cells. In Table 2 the correlation between the genotype and the inflammatory infiltrate burden is reported. The higher presence of 249V/V (52%) in AMI group as compared to controls is correlated with the presence of a consistent T cell infiltrate expressing CX3CR1 (*P* < 0.01), confirming the Th1 polarized subpopulation recruitment in the plaque.

## 4. Correlation among AMI, AMI Survivors and NCC Genotype

The genotype analysis has been extended to a cohort of individuals survived at a first myocardial infarction (*n* = 16), in order to compare the presence of the allele variant with the occurrence and the outcome of the acute event. 

We found that the 52% of AMI patients (13/25) displayed the V249 allele in homozygosis, as compared with the 6% of the AMI survivors (1/16) and the 9% of controls (2/22) (Table 3).

The multivariate analysis of each V249I polymorphism, adjusted for clinical data in the three study groups, showed that the V/V249 haplotype was significantly associated only with the fatal AMI (*P* = 0.001) but not with all the major risk factors.

On the other hand, the I249 variant allele was expressed in homozygosis only in the 8% of the AMI patients (2/25) (Table 3), as compared with the 36% of controls (8/22) and the 31% of AMI survivors (5/16).

The analysis of T280M polymorphism displayed the presence of the M280 variant allele in homozygosis in the 4% of controls (1/22), as compared with AMI and survivors patients, which showed no presence of this allele in homozygosis. The presence of M280 allele in homozygosis was always associated with the I249 allele.

In summary, the significant higher presence of the variant allele I249 in homo- and heterozygosis found in controls (91%) and in AMI survivors (94%), with respect to the patients died of AMI (48%), showed the relevance of this polymorphism both in the onset and outcome of acute myocardial infarction, confirming the correlation between CX3CR1 genotype and prognosis of the acute event.

## 5. Discussion

Epidemiological and clinical studies have shown that the CX3CR1 receptor polymorphisms are linked with the risk of coronary artery diseases [14, 15].

In particular, the presence of CX3CR1 V249 and T280 single nucleotide polymorphisms codify for a fractalkine receptor that is overexpressed by activated inflammatory cells and with higher binding affinity for fractalkine, its natural ligand [16, 20]. 

The genotyping analysis of the subjects enrolled in the present study demonstrated for the first time a direct correlation between CX3CR1 V249I and T280M polymorphisms and the occurrence of acute fatal myocardial infarction. A striking link between genotype and prognosis of the acute event was suggested by the observation of AMI survivors genotypes. Notably, the in situ CX3CR1 high expression in AMI patients correlates with the presence of V249 polymorphism in homo- or heterozygosis. Conversely, the genotype analysis of controls (with no CVD history) demonstrated the presence of I249 polymorphism in homozygosis.

Indeed, CX3CR1 V249/T280 polymorphisms in homozygosis or heterozygosis have been demonstrated in 92% of the AMI patients analyzed (mean age: 70 years old). In particular, 52% of the AMI patients examined were homozygous for CX3CR1 V249, whereas the analysis of the genotype of the control subjects (mean age: 73.7 years old) demonstrated the presence of CX3CR1 V249 polymorphism in homozygosis only in the 9% of cases. The findings of the high percentage of I/I249 (36%) and M/M280 (4%) in subjects died of no cardiac causes suggest a possible link between CX3CR1 polymorphism, T cell recruitment, and acute event. Moreover, the genotype observations on survivors group suggest that the presence of I249 allele is relevant not only for the occurrence but also in the outcome of the acute event.

There is evidence of the influence of CX3CR1 variable expression and function on T cells recruitment efficiency [14, 21, 22].

Studies on CX3CR1 null mice, crossed into apoE ^−^/^−^ background [12], demonstrated that, although CX3CL1 was strongly expressed in smooth muscle cells in the lesion, the lack of the CX3CR1 receptor was related to a significant reduction in macrophages and T cells recruitment within the vessel wall, associated with a reduction of atherosclerosis.

In present work, we also show a close association between CX3CR1 genotypes and coronary plaques T cell inflammation, that is, a relevant character of vulnerable plaques according to data previously published [1, 2].

Indeed, coronary plaques of patients with CX3CR1 I249 allele displayed a weaker T cell inflammation as compared to individuals homozygous for the CX3CR1 V249 allele.

In particular, we show that CX3CR1 is highly expressed by the majority of T cells infiltrating IRA plaques of 22 out of 25 AMI patients.

It is worth noting that the two patients showing a low expression of CX3CR1 were CX3CR1 I249 homozygou and their plaques were infiltrated by B lymphocytes, suggesting a different pathogenesis for AMI.

Conversely, immunostaining for CX3CR1 was negative or faint positive in plaques of controls, where the T lymphocytes infiltrate was lower as compared with AMI patients.

Therefore, it can be assumed that the high expression of CX3CR1 together with the in situ expression of fractalkine (CX3CL1) correlates with the increased recruitment of T lymphocytes deeply infiltrating in the plaques of the AMI patients.

The special feature of fractalkine among other members of the chemokine family is due to the fact that it is expressed both as soluble and membrane-bound form on the surface of inflamed endothelium. Interestingly, whereas soluble fractalkine (CX3CL1) was reported to recruit lymphocytes and monocytes, immobilized forms of CX3CL1 have been shown to directly mediate the rapid capture and firm adhesion of leukocytes expressing its receptor CX3CR1 under physiological flow conditions [23]. The direct binding of the chemokine to CX3CR1 does not require the upregulation and activation of integrins, suggesting that fractalkine and CX3CR1 mediate a novel pathway for leukocytes trafficking [24]. In addition to the intrinsic adhesive function of fractalkine, this chemokine can also transduce signals through G proteins that enhance the avidity of integrin binding to its ligands for leukocytes recruitment [25, 26].

The coexpression of CD3, IFN-gamma, and CX3CR1 on T cell population found in AMI plaques compared to controls demonstrates the in situ activation of inflammatory cells pointing out the probable involvement of a recent immune Th1 response in plaque destabilization and rupture. Moreover, the data obtained suggest that the susceptibility to vascular inflammation is correlated with the amplified recruitment of Th1 polarized cells expressing the high or low affinity CX3CR1 receptor. The findings revealing CX3CR1 expression by 80% of T cell population along with the presence of fractalkine in IRA plaques suggest that these two events are both likely to play a major role in determining plaque vulnerability by orchestrating Th1 cells recruitment. 

In agreement with previous clinical and epidemiological studies, our data demonstrate a close association between V249I and T280M polymorphisms, coronary plaque vulnerability, and AMI, confirming that the subjects with I249 allele display a low level of T cell inflammation and a reduced risk of developing vulnerable plaques and they have a decreased incidence of acute events (probably due to a lower CX3CR1 binding affinity) [27]. The comparative analysis of the different CXCR3 polymorphisms and the inflammatory infiltrate shows a significant difference between fatal AMI and NCC. A study limitation is the lacking in the comparative analysis of the coronary surgical samples from AMI survivors. These patients in fact underwent a ventricular restoration for a previous nonfatal AMI event. Despite the above limitations, our study provides lines of evidence that the I249 allele could be strongly involved in the protection from fatal AMI, with these association being independent of the major “conventional” cardiovascular risk factors. Further studies will be addressed with a prospective massive scale population study to validate this assumption. 

In conclusion, CX3CR1 genotype could be considered as a new marker to stratify the population according to fatal AMI risk. Moreover, CX3CR1 is likely to amplify the Th1 immune response, directly related to plaque vulnerability, irrespectively of the causal agents (i.e. ox-LDL or Chlamydia), thus, standing out as a potential target in fatal AMI prevention. 

## Figures and Tables

**Table 1:** Clinical and surgical data of patients.

	AMI* group (25 pts)	NCC* group (22 pts)	SVR* group (16 pts)
Clinical data			
Age (yr ± SE)	70.5 ± 1.8	73.7 ± 2.8	65 ± 2.7
Male	17 (68)	15 (68)	13 (81)
Hypertension	18 (72)	14 (64)	11 (69)
Diabetes	13 (52)	10 (45)	7 (44)
Previous smoking	13 (52)	11 (50)	7 (44)
Hyperlipidemia	14 (56)	10 (45)	8 (50)
Previous MI (%)	0	0	16 (100)
Previous PTCA	0	0	5 (31)
Autoptic data: causes of death			
Acute MI:	25 (100)	0	
Anterior wall	13 (52)	0	
Posterolateral wall	7 (28)	0	
Posterior wall	5 (20)	0	
Bronchopneumonia	0	14 (64)	
Pulmonary embolism	0	5 (23)	
Intestinal hemorrhage	0	3 (14)	
Surgical data			
Mean time from MI to Op (mo ± SE)			51 ± 9
EF (%± SE)			30 ± 2.5
LVEDV (mL ± SE)			183 ± 10.2
LVESV (mL ± SE)			129 ± 10.1
CABG			15 (94)
LIMA grafts			15 (94)
No. of total anastomosis (±SE)			2.9 ± 0.3

*AMI: acute myocardial infarction patients; NCC: subjects dead for noncardiac causes; SVR: patients survived to a first AMI.

Data are absolute numbers (percentage).

Abbreviations: CABG: coronary artery bypass graft; EF: ejection fraction; LIMA: left internal mammary artery; LVEDV: left ventricle end-diastolic volume; LVESV: left ventricle end-systolic volume; MI: myocardial infarction; PTCA: percutaneous transluminal coronary angioplasty; SE: standard error.

**Table 2:** Correlation between inflammatory infiltrate and CX3CR1 polymorphism^§^.

	AMI			NCC		
	V/V249 13/25 (52)*	V/I249 10/25 (40)	I/I249 2/25 (8)	V/V249 2/22 (9)	V/I249 12/22 (55)	I/I249 8/22 (36)
CD3						
Neg./weak	0	0	1	1	7	7
Pos. moderate	0	1	1	1	5	0
Strong	13	9	0	0	0	0
CD20						
Neg./weak	12	9	0	2	12	7
Pos. moderate	1	1	0	0	0	0
Strong	0	0	2	0	0	0
CXCL1						
Neg./weak	0	0	0	1	12	7
Pos. moderate	1	1	2	1	0	0
Strong	12	9	0	0	0	0
CXCR1						
Neg./weak	0	0	1	1	7	7
Pos. moderate	0	1	1	0	5	0
Strong	13	9	0	1	0	0

^§^The table shows the IHC results in the coronary plaque of AMI and control patients (NCC), correlated to the presence of CX3CR1 V249I polymorphism in the same groups. *In parentheses, the percentage of patients was reported.

**Table 3:** Analysis of CX3CR1 polymorphisms in patients and control groups.

	AMI (*n* = 25)	SVR (*n* = 16)	NCC (*n* = 22)
V249I			
V/V	13 (52)	1 (6)	2 (9)
V/I	10 (40)	10 (63)	12 (55)
I/I	2 (8)	5 (31)	8 (36)
	*P* = 0.003	*P* = 0.003	*P* = 0.006
T280M			
T/T	21 (84)	9 (56)	14 (64)
T/M	4 (16)	7 (44)	7 (32)
M/M	0	0	1 (4)
	*P* = 0.001	*P* = 0.002	*P* = 0.001

Data are absolute numbers (percentage).

Abbreviations: AMI: acute myocardial infarction; SVR: patients survived to a first AMI; NCC: subjects dead for noncardiac causes.

The statistical significance of different polymorphisms present in the single groups was identified by Fisher's exact test.
